# miR‐1, miR‐499 and miR‐208 are sensitive markers to diagnose sudden death due to early acute myocardial infarction

**DOI:** 10.1111/jcmm.14463

**Published:** 2019-06-26

**Authors:** Enrica Pinchi, Paola Frati, Mariarosaria Aromatario, Luigi Cipolloni, Matteo Fabbri, Raffaele La Russa, Aniello Maiese, Margherita Neri, Alessandro Santurro, Matteo Scopetti, Rocco Valerio Viola, Emanuela Turillazzi, Vittorio Fineschi

**Affiliations:** ^1^ Department of Anatomical, Histological, Forensic and Orthopaedic Sciences Sapienza University of Rome Rome Italy; ^2^ IRCSS Neuromed Mediterranean Neurological Institute Pozzilli Italy; ^3^ Department of Morphology, Experimental Medicine and Surgery University of Ferrara Ferrara Italy; ^4^ Institute of Legal Medicine, Department of Surgical, Medical and Molecular Pathology and Critical Care Medicine University of Pisa Pisa Italy

**Keywords:** acute myocardial infarction, miR‐1, miR‐208, miR‐499, sudden cardiac death

## Abstract

MicroRNAs (miRNAs) are strongly up‐regulated under pathological stress and in a wide range of diseases. In recent years, miRNAs are under investigation for their potential use as biomarkers in cardiovascular diseases. We investigate whether specific cardio‐miRNAs are overexpressed in heart samples from subjects deceased for acute myocardial infarction (AMI) or sudden cardiac death (SCD), and whether miRNA could help differentiate between them. Forty four cases of death due to cardiovascular disease were selected, respectively, 19 cases categorized as AMI and 25 as SCD. Eighteen cases of traumatic death without pathological cardiac involvement were selected as control. Immunohistochemical investigation was performed for CD15, IL‐15, Cx43, MCP‐1, tryptase, troponin C and troponin I. Reverse transcription and quantitative real‐time PCR were performed for miR‐1, miR‐133, miR‐208 and miR‐499. In AMI group, stronger immunoreaction for the CD15, IL‐15 and MCP‐1 antibodies was detectable compared with SCD and control. Cx43 showed a negative reaction with respect to the other groups. Real‐time PCR results showed a down‐regulation of all miRNAs in the AMI group compared with SCD and control. The selected miRNAs presented high accuracy in discriminating SCD from AMI (miR‐1 and miR‐499) and AMI from control (miR‐208) representing a potential aid for both clinicians and pathologists for differential diagnosis.

## 1 INTRODUCTION

Cardiovascular diseases are among the leading causes of death worldwide and account for approximatively 17 million deaths every year and about 25% are sudden cardiac deaths (SCD).^1^ Data from the United States estimated that 300,000‐400,000 deaths annually are SCD.^2^ Similar data are reported in Europe.^3^

However, the true incidence of SCD remains undetermined widely ranging in the available estimates^4^ due to several factors. One is the persistent lack of consensus on its definition, as systematically reviewed by Kong et al^4^ who found a great number of studies giving different definitions of SCD grounded, alternately, on time constraints, geographical location of the event, and, finally, on the aetiology of the event SCD. Among others, SCD may be defined as unexpected, non‐traumatic death occurring within one hour of the onset of new or worsening symptoms (witnessed arrest) or, if unwitnessed, within 24 hours of last being seen alive.^5^ Further difficulties in exactly quantifying the incidence of SCD derive from the fact that most of the available estimates are based on retrospective death certificate‐based methodology^4^: Death certificates, vital statistics and census data can misclassify and overestimate SCD.^6^

Finally, a further confounding factor is the fact that SCD can be challenging to recognize during the autopsy. Pathologists often refer to sudden unexplained death when an exhaustive post‐mortem examination fails to determine a conclusive cause of death.^7^ Many SCD cases still remain unexplained even when conventional autopsy and macroscopic and histological investigations are thoroughly performed; consequently, the search for and identification of the cause of death still remain a heavy challenge for pathologists. In recent years, structured autopsy procedures^8^ and technological advances have facilitated the post‐mortem study of SCD. Post‐mortem imaging,^9^, ^10^ laboratory, genetic and immunohistochemical investigations have inalienably helped pathologists in the post‐mortem assessment of SCD cases. Timing of injury is a key element in forensic pathology showing rising interest in implementing immunohistochemistry essays in order to corroborate qualitative evidence with semiquantitative parameters. Not surprisingly, then, different immunohistochemical markers have been tested for their predictive value in timing the onset of acute myocardial infarction (AMI). So, we need to investigate the pathology of myocardial infarction as a cause of sudden cardiovascular death. Chronological evaluation needs the analysis of parameters within a histological specimen such as any measurement of the number and size of cellular expressions/modifications. The criteria to determine the chronological changes in AMI are supported by many technologies and methodologies available to pathologists, but are unfortunately very rarely applied in daily practice.

Furthermore, genetic investigations have notably increased diagnostic accuracy in SCD cases^11^, ^12^ since genome‐wide association studies have demonstrated numerous variations within DNA sequence leading to increased risk of cardiovascular deaths (CVDs).^13^ However, a certain quote of CVD genetic risk remains unknown (“missing heritability”) and the hypothesis that epigenetic mechanisms could be partially responsible for the “remaining” genetic risk of CVD is increasingly strengthened.^14^ Consequently, for both clinicians and pathologists, the search for reliable markers of SCD still remains challenging.^15^

In this context, microRNAs (miRNAs) generated enormous enthusiasm for their potential use as biomarkers of several cardiovascular diseases.^16^, ^17^ MiRNAs are a class of small non‐coding RNAs of ~22 nt that suppress gene expression by hybridizing to the 3' untranslated region of messenger RNA (mRNA), promoting mRNA degradation or disrupting translation, thus acting as post‐transcriptional regulators, repressing, or completely silencing, protein translation. Several miRNAs are strongly up‐regulated during pathological stress, and they appear to be aberrantly expressed in blood plasma or serum during the course of many diseases.^18^

Among the plethora of miRNAs progressively annotated (about 1500‐2000 human miRNAs have been identified),^19^ some miRNAs have been demonstrated to play a significant role in cardiogenesis, heart function and pathology,^20^, ^21^ thus contributing to the progression of cardiovascular diseases such as cardiac hypertrophy and fibrosis and myocardial infarction.^22^, ^23^ Cardiac miRNAs such as miR‐1, miR‐133a, miR‐208a/b and miR‐499 are abundantly expressed in the myocardium and are present, stable and detectable in the circulation in different cardiovascular events,^15^, ^16^, ^17^, ^18^, ^19^, ^20^, ^21^, ^22^, ^23^, ^24^ such as early after myocardial infarction.^25^ MiRNAs have also been explored for cardiovascular risk stratification,^26^ thus assuming an increasingly important role as potential cardiovascular biomarkers.^27^

Given that specific miRNAs are actively secreted by cardiomyocytes and that cardiomyocyte‐derived miRNAs can be found in circulation in various cardiovascular acute events such as AMI and SCD, in the present study, we investigated whether specific cardio‐miRNAs are overexpressed in cardiac tissue samples of subjects deceased for AMI or SCD and whether these different cardiac diseases could be differentiated by the miRNA expressed in cardiac tissue samples taken at autopsy.

## 2 MATERIALS AND METHODS

The clinical data and autopsy records of the cardiovascular death (CVD) autopsies performed at the Department of Anatomical, Histological, Forensic and Orthopaedic Sciences—Sapienza University of Rome and the University of Pisa (Italy) over the period 2013‐2017 were evaluated. We selected 44 cases of subjects who died for CVD. We selected only cases with a well‐defined clinical course (clinical symptoms, ECG and laboratory data) and with post‐mortem confirmed CVD diagnosis. Based on the available clinical and laboratory data, and on the autoptic, histopathological, and immunohistochemical investigation, 19 subjects were classified as having an AMI.

AMI diagnosis was determined by a cardiologist based on clinical history, physical examination, electrocardiography and cardiac markers. In all cases, a 12‐lead ECG was performed within 10 minutes of arrival into the emergency department. If the standard leads were inconclusive and the patient had signs or symptoms suggestive of ongoing myocardial ischaemia, additional leads were recorded since left circumflex artery occlusion or right ventricular MI could be detected only in V7‐V9 and V3R and V4R, respectively. In patients presenting with cardiac arrest or haemodynamic instability of presumed cardiovascular origin, echocardiography was performed immediately following a 12‐lead ECG.

STEMI was defined by the presence of diagnostic STE defined as new STE at the J point in at least two contiguous leads ≥2 mm (0.2 mV) in men or ≥1.5 mm (0.15 mV) in women in leads V2‐V3 and/or of ≥1 mm (0.1 mV) in other contiguous chest or limb leads.^28^ Findings as transient ST elevation, ST depression or new T wave inversions were considered suggestive of NSTEMI.

Cardiac troponin (cTn) and other biomarkers (CKMB) were measured on first assessment and, depending on the patient's survival, repeated 3‐6 hours later. An elevated high sensitivity cTn value (>99th percentile URL) was considered diagnostic for AMI. If a cTn assay was not available, an increased CKMB value above the 99th percentile URL was designated as the decision level for the diagnosis of MI.^29^

Twenty five subjects who had deceased suddenly, unexpectedly and with no pathogenic findings after thorough macro—and histopathological post‐mortem investigations were categorized as SCD. Survival time ranged from 4 to 6 hours to no more than 12 hours from the abrupt onset of typical symptoms. Control group consisted of 18 cases of traumatic death (survival limited to 12 hours) without cardiac alterations (Table 1). In all cases, toxicology screening was negative for alcohol and drugs of abuse. In all cases, the myocardial samples (standard seven specimens and additional samples taken from macroscopically altered areas) were re‐examined histologically to confirm the diagnosis of the cause of death and total RNA, including miRNAs, was extracted from these formalin‐fixed paraffin‐embedded (FFPE) tissue blocks.

**Table 1:** Demographic characteristics, clinical data and biomarkers results of subjects under analysis

Variable	AMI subjects (n = 19)	SCD subjects (n = 25)	Control group (n = 18)
Age (y)			
Mean	63.8	60.1	66.4
Range	47‐79	44‐76	45‐73
Gender			
Male	13	15	12
Female	6	10	6
Type of AMI			
STEMI	10 (53%)	—	—
NSTEMI	9 (47%)	—	—
Clinical symptoms			
Persistent chest pain	15 (79%)	5 (20%)	—
Heart failure	7 (37%)	—	—
Other symptoms (epigastric pain, indigestion‐like symptoms, isolated dyspnoea, etc)	8 (42%)	10 (40%)	—
Therapy			
Titrated intravenous (iv) opioids	9 (47%)	—	3 (17%)
Mild tranquillizer (benzodiazepine)	10 (53%)	—	—
Primary PCI	6 (32%)	—	—
Fibrin—specific agent (ie tenecteplase, alteplase or reteplase)	4 (21%)	—	—
Intravenous or sublingual nitrates	9 (47%)	—	—
Beta blockers	7 (37%)	—	—
Anticoagulation	9 (47%)	—	—
Catecholamine	2 (11%)	—	14 (78%)
Biomarkers			
cTN (above the 99th percentile)	19 (100%)	—	—
CKMB (above the 99th percentile)	19 (100%)	—	—

Data processing complies with the general authorization for scientific research purposes granted by the Italian Data Protection Authority (1 March 2012 as published in Italy's Official Journal no. 72 dated 26 March 2012) since the data do not entail any significant personalized impact on data subjects. Approval by an institutional and/or licensing committee is not required since experimental protocols are not applied in the study. All cases are judicial and come from autopsies ordered by local prosecutors to clarify the exact cause of death.

### 2.1 Immunohistochemistry

As previously reported,^30^ cardiac samples were investigated by immunohistochemistry utilizing a panel of antibodies: anti‐CD15 (DAKO, Copenhagen, Denmark), anti‐IL‐15 (R&D Systems, Minneapolis, MN), anti‐MCP‐1 (Santa Cruz, CA), anti‐tryptase (Novus Biologicals, Littleton, CO), anti‐Cx43 antibody (Sigma‐Aldrich, St. Louis, MO) and TUNEL assay. Cellular antigen troponin C (Novocastra Leica Biosystems GmbH, Nussloch, Germany) and troponin I (Thermo Fisher Scientific, Fremont, CA), early markers of myocardial necrosis, were also investigated.^31^, ^32^, ^33^ Four‐micrometre‐thick paraffin sections were mounted on slides covered with 3‐aminopropyl‐triethoxysilan (Fluka, Buchs, Switzerland). A pre‐treatment was performed to enhance antigen retrieval and membrane permeability to antibodies. In particular, anti‐CD 15, MCP‐1, IL‐15, Cx43, troponin C and troponin I were heated at boiling water in 0.25 mol/L EDTA buffer. The dilution ratio of primary antibodies was 1:50 for CD 15, 1:100 for IL‐15, 1:200 for Cx43, 1:500 for MCP‐1, 1:1000 for tryptase, 1:3000 for troponin I and 1:6000 for troponin C. Incubation occurred for 120 minutes at 20°C. For TUNEL assays (Chemicon, Temecula, CA), the sections were covered with the TdT enzyme, diluted in a ratio of 30% in reaction buffer (Apotag Plus Peroxidase In Situ Apoptosis Detection Kit; Chemicon) and incubated for 60 minutes at 38°C. The detection system utilized was the LSAB + kit (Dako, Copenhagen, Denmark), a refined avidin‐biotin technique in which a biotinylated secondary antibody reacts with several peroxidase‐conjugated streptavidin molecules. The positive reaction was visualized by 3.3‐diaminobenzidine (DAB) peroxidation, according to standard methods. The sections were counterstained with Mayer's haematoxylin, dehydrated, coverslipped and observed in a Leica DM4000B optical microscope (Leica, Cambridge, UK).

A semiquantitative analysis was blindly performed by two investigators. Results were graded as follows: 0 = not expressed; +, isolated and disseminated expression; ++, expression in widespread foci; and +++, widespread expression. Two examiners performed all measurements at the same magnification of image (10×). A third researcher evaluated blindly the results to weigh the histological evidence. The samples were also viewed under a confocal microscope, thus allowing a three‐dimensional reconstruction (True Confocal Scanner, Leica Biosystems GmbH TCS SPE).

### 2.2 RNA extraction protocols

Usually, total RNA from FFPE tissue blocks is considered unsuitable for gene expression assays since fixation and embedding procedures cause chemical modifications which strongly affect nucleic acid integrity. Thus, mRNA obtained from FFPE materials is fragmented (fewer than 300 bases), contains cross‐linkages with proteins and several chemical modifications (formation of Schiff bases, methylol and stable methylene derivates) which hinder downstream applications. On the contrary, miRNA molecules are less prone than mRNAs to degradation and chemical modification and easier to recover from FFPE samples, due to their very small size and close association with several large protein complexes. The suitability of FFPE tissue for real‐time RT‐qPCR analysis of miRNAs has been reported by several authors, although this approach has been more often performed on surgical rather than autoptic FFPE samples. Despite this, miRNAs are less impaired by post‐mortem decay compared to other RNAs. This behaviour could be attributable to their size (about 22 nt) and association with protein complexes which prevent degradation. All these characteristics confer to miRNAs high forensic relevance.^34^

In this study, total RNA, including miRNAs, was extracted from FFPE tissue blocks using the High Pure miRNA isolation kit (Roche Basilea, Switzerland). For each sample, 5 paraffin‐embedded sections 7‐μm‐thick were obtained. Deparaffinization was carried out by incubating the sections at 60°C for 20 minutes and soaking in xylene for 15 minutes for two changes. The rehydration of sections was performed gradually through graded alcohols. The tissues were carefully collected in an autoclaved plastic microtube (1.5 cm). A mix of 100 μL of paraffin tissue lysis buffer, 16 μL of 10% SDS and 40 μL of proteinase K was used to lyse the tissues. Incubation was performed at 56°C overnight. When all tissue fragments were dissolved, 325 μL of binding buffer and 325 μL of binding enhancer were added and the lysates were loaded onto the High Pure Filter Tube. A brief centrifugation was carried on to allow RNA to be adsorbed onto the filter membrane followed by two washing steps. Purified RNA was eluted using 50 μL of elution buffer. RNA concentrations were investigated with NanoDrop (Thermo Scientific Darmstadt, Germany).

### 2.3 Reverse transcription and quantitative real‐time PCR

Reverse transcription and quantitative real‐time PCR were performed for the following miRNAs: miR‐1, miR‐133, miR‐208 and miR‐499.

Each sample was reverse‐transcribed with TaqMan MicroRNA Reverse Transcription Kit (Applied Biosystems Darmstadt, Germany) according to the manufacturer's protocol. Total RNA was converted into cDNA by incubation with reverse transcriptase (RT) at 16°C for 30 minutes, 42°C for 30 minutes, 85°C for 5 minutes and 4°C. The same amount of RNA (10 ng) for each sample was added to the RT reaction mix in a total volume of 15 μL, as recommended by the manufacturer (Applied Biosystems, Protocol 4367038 Rev. E).

For PCR assays, TaqMan MiRNA Assay (Applied Biosystems, Darmstadt, Germany) was used. RT‐qPCR was performed with Stratagene Mx3005p Real‐Time PCR equipment with 96‐well optical reaction plates. PCR mixture (total volume 20 μL) contained 1.33 mL of RT product, 10 μL of TaqMan 2× Universal PCR Master Mix and 1 μL of the specific TaqMan MicroRNA Assay (20×) containing probes for the miRNA of interest (Applied Biosystems, Darmstadt, Germany). The mixture was initially incubated at 95°C for 10 minutes, followed by 40 cycles of 95°C for 15 seconds and 60°C for 60 seconds. Samples were run in duplicate, and results were averaged. RNU48 (U48) and U6 were used to normalize data.

The −∆Ct method was used to calculate the relative expression of the target genes as follows:

$$-\Delta {\text{Ct}}_{\text{miRNA}}=-\left({\text{Ct}}_{\text{miRNA}}-{\text{Ct}}_{\text{housekeeping}}\right)$$

Sequences were reviewed on miRNA database at http://www.mirbase.org (Table 2).

**Table 2:** Sequences of the miRNAs analysed

miRNA Name	Identification	Accession number	Primer sequences
miR‐1‐3p	hsa‐miR‐1	MIMAT0000416	UGGAAUGUAAAGAAGUAUGUAU
miR‐208a‐3p	hsa‐miR‐208	MIMAT0000241	AUAAGACGAGCAAAAAGCUUGU
miR‐133a‐3p	hsa‐miR‐133	MIMAT0000427	UUUGGGUCCCCUUCAACCAGCUG
miR‐499a‐5p	hsa‐miR‐499	MIMAT0002870	UUAAGACUUGCAGUGAUGUUU

### 2.4 Statistical analysis

Data were expressed as the mean ± SD from at least three independent experiments. Statistical analysis was performed using Student's *t* test. The diagnostic power of miR‐1, miR‐208, miR‐133 and miR‐499 was evaluated through receiver operating characteristic (ROC) analysis. Areas under the curve (AUCs) were calculated. All statistical analyses were performed using GraphPad Prism software (GraphPad software). *P* < 0.05 was considered statistically significant.^35^ A semiquantitative analysis of the immunohistochemical results and gradation of the immunohistochemical reaction were rendered with an ordinal scale and the median value reported. Variance for the non‐parametric data between groups was analysed by the Kruskal‐Wallis test. Differences found to be significant led to the analysis of the unmatched groups with a Dunn's multiple comparison post hoc test. The significance level was set to 5% (SPSS ver. 16.01 for Windows—SPSS Inc, Chicago USA).

## 3 RESULTS

### 3.1 Immunohistochemistry

**Figure 1:**
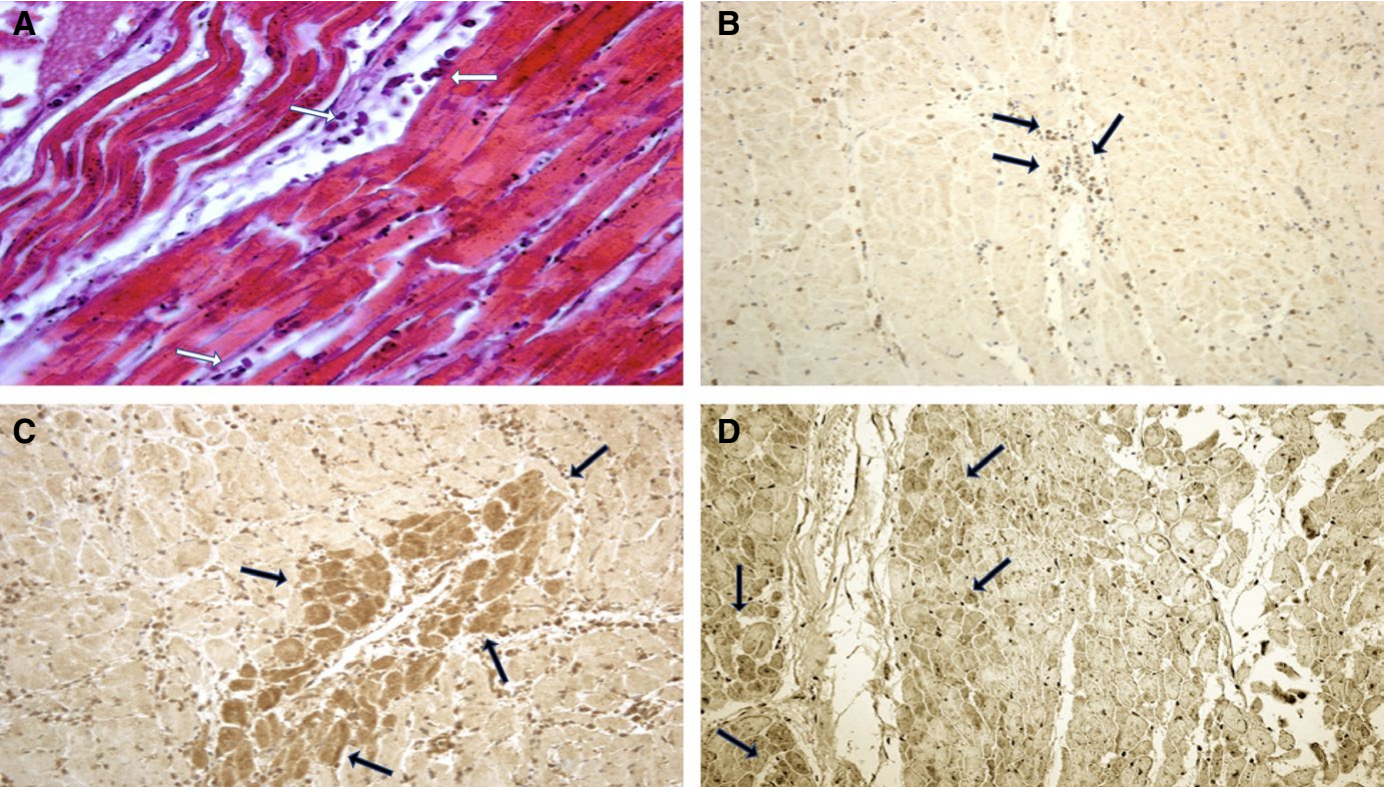
Histological and immunohistochemical investigation of group A acute myocardial infarction cardiac samples. (A), Haematoxylin and eosin (H&E) staining: neutrophils and monocytes in vessels (arrows) at the periphery of the necrotic zone along with infiltration into the ischaemic tissue (×100); (B) CD‐15: massive and diffuse positive reaction (arrows) in several myocardial areas (×40); (C) IL‐15: intense expression (arrows) in the microscopic fields in which the margination of circulating inflammatory cells was detectable (×60); (D) MCP‐1: diffuse positivity (arrows) of the immunoreaction within the ischaemic tissue (×60)

Differences in the myocardial structure were found (Table 3). In the group of SCD, mild myofibre eosinophilia and elongation of sarcomeres and nuclei were fairly evident as well as prominent contraction band necrosis. Polymorphonuclear (PMN) margination was not observed. In the same cases, a negative immunoreaction of CD15, IL‐15 and MCP‐1 matched by the negative markers of necrosis (cellular antigen troponin I and C) was present. Immunolabelled Cx43‐positive granules were small, irregular and scattered. In the group of AMI, the presence of PMN leucocytes (neutrophils and monocytes) was detectable in vessels marginal to the periphery of the necrotic zone; infiltration of the same cells into the ischaemic tissue was concomitant. PMNs crowded along a line between infiltrated and non‐infiltrated necrotic myocardium in large areas of necrosis were visible. Immunohistochemistry confirmed the nature of the inflammatory cells. A stronger immunopositivity for the CD15, IL‐15 and MCP‐1 antibodies was observed colocalized with the margination of circulating inflammatory cells and central part of the AMI (Figure 1). Cx43 immunohistochemically positive reaction demonstrated few brown granules in the AMI group, rarely expressed at the intercellular junctions.

**Table 3:** Semiquantitative evaluation and statistical analysis of the immunohistochemical findings and gradation of the immunopositivity reaction in the heart samples

Antibody	Group A	Group B	Group C	Significant levels	Significance levels^b^
	AMI^a^	SCD^a^	C^a^		
CD‐15	+++	—	—	A vs C	***
				B vs C	ns
				A vs B	***
IL‐15	++	—	—	A vs C	**
				B vs C	ns
				A vs B	**
MCP‐1	++	—	—	A vs C	**
				B vs C	ns
				A vs B	**
Troponin C	+++	—	—	A vs C	***
				B vs C	ns
				A vs B	***
Troponin I	+++	—	—	A vs C	***
				B vs C	ns
				A vs B	***
Tryptase	+++	—	—	A vs C	***
				B vs C	ns
				A vs B	***
Cx43	+/−	+	+++	A vs C	***
				B vs C	**
				A vs B	ns

a Intensity of immunopositivity was assessed semiquantitatively in the scale 0‐4 as follows: ‐, not expressed; +, isolated and disseminated expression; ++, expression in widespread foci; and +++, widespread expression.

b ns: *P* > 0.05; *P < 0.05; **P < 0.01; ***P < 0.001.

**Figure 2:**
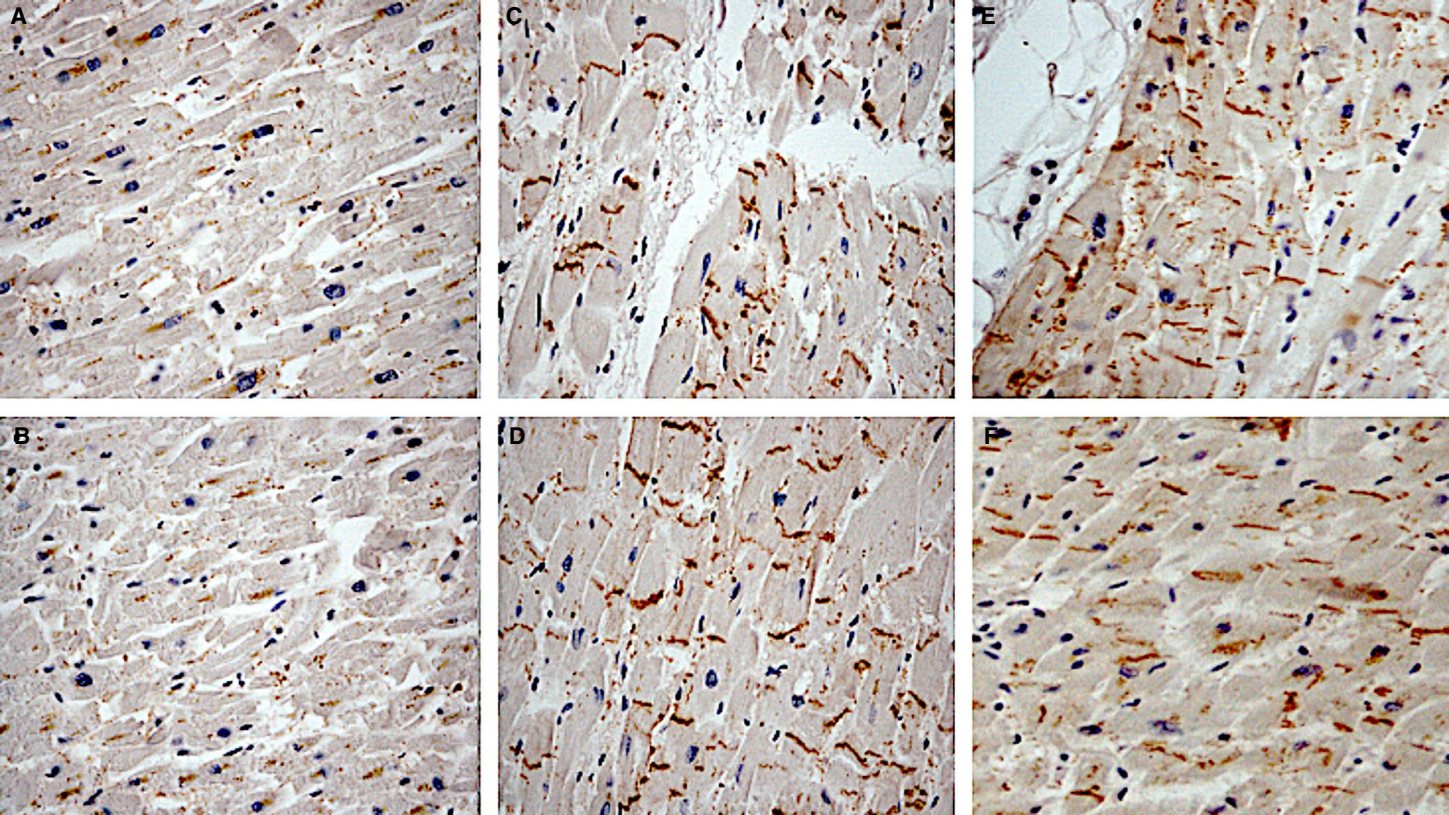
Cx43 immunohistochemically positive reaction demonstrated few brown granules in the acute myocardial infarction group, rarely expressed at the intercellular junctions (×100) (A,B). In the group of sudden cardiac death, immunolabelled Cx43‐positive granules were small, irregular and scattered (×100) (C,D). The control group demonstrated Cx43 granules distributed in the intercalated disc and also in the cellular bridges between the myocardial cells (×100); E,F)

The control group demonstrated CD15, IL‐15 and MCP‐1 negative reactions. Cx43 granules were distributed in the intercalated disc and also in the cellular bridges between the myocardial cells (Figure 2).

### 3.2 miRNA

**Figure 3:**
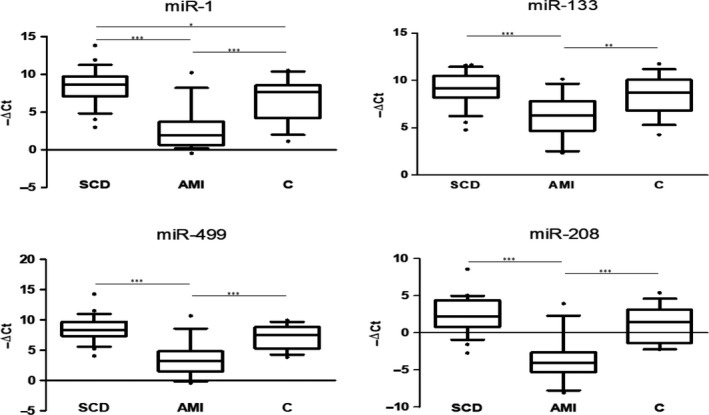
Expression levels expressed as −ΔCt (median with range and interquartile range) of selected miRNAs. *P = 0.05; **P = 0.01; ***P = 0.001

Real‐time PCR results (Figure 3) showed a down‐regulation of all miRNAs investigated in the AMI group compared with both SCD and control group. Furthermore, significant differences in the expression of miR‐1 were found between SCD and controls, being up‐regulated in SCD. Notably, miRNAs under analysis were not able to discriminate cases of SCD from subjects deceased for other causes, with the exception of miR‐1 that was higher in SCD cases compared to the other two groups.

**Figure 4:**
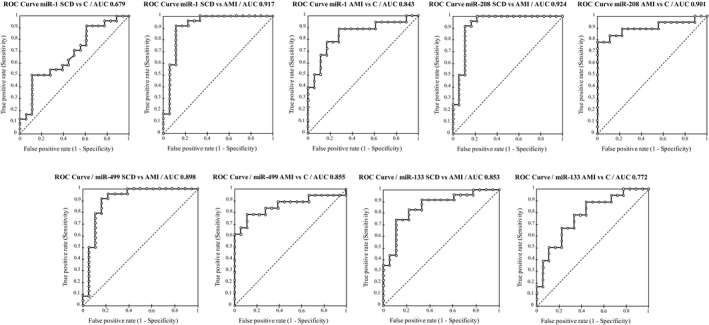
Receiver operating characteristic (ROC) analysis of selected miRNAs

In order to estimate the diagnostic accuracy of the selected miRNAs, ROC curve analysis was performed (Figure 4).

Selected miRNAs obtained the best result in discriminating the SCD from AMI cases reaching the highest AUC values (Table 4). Nonetheless, good results were obtained comparing AMI vs C.

**Table 4:** Receiver operating characteristic analysis of selected miRNAs

	Test threshold	Sensitivity	LB (95%)	UB (95%)	Specificity	LB (95%)	UB (95%)	PPV	NPV	TP	TN	FP	FN	Sensitivity + Specificity	Accuracy
miR‐1 AMI vs C	3.470	0.778	0.541	0.913	0.833	0.598	0.948	0.824	0.789	14	15	3	4	1.611	0.806
miR‐1 SCD vs AMI	5.620	**0.917**	0.728	0.987	0.889	0.657	0.979	0.917	0.889	22	16	2	2	**1.806**	0.905
miR‐1 SCD vs C	8.770	0.500	0.315	0.685	0.889	0.657	0.979	0.857	0.571	12	16	2	12	1.389	0.667
miR‐208 AMI vs C	−2.790	0.778	0.541	0.913	**1.000**	0.789	1.000	1.000	0.818	14	18	0	4	1.778	0.889
miR‐208 SCD vs AMI	−0.350	0.917	0.728	0.987	0.889	0.657	0.979	0.917	0.889	22	16	2	2	**1.806**	0.905
miR‐499 AMI vs C	4.570	0.778	0.541	0.913	0.889	0.657	0.979	0.875	0.800	14	16	2	4	1.667	0.833
miR‐499 SDC vs AMI	5.880	**0.917**	0.728	0.987	0.833	0.598	0.948	0.880	0.882	22	15	3	2	1.750	0.881
miR‐133 AMI vs C	6.730	0.667	0.435	0.837	0.778	0.541	0.913	0.750	0.700	12	14	4	6	1.444	0.722
miR‐133 SCD vs AMI	8.520	0.739	0.531	0.876	0.889	0.657	0.979	0.895	0.727	17	16	2	6	1.628	0.805

Test threshold, sensitivity (corresponds to the rate of positive cases that are well diagnosed by the test), specificity (corresponds to the rate of negatives cases that are well diagnosed by the test).

Numbers in bold indicate the highest values of combined sensitivity + specificity in the comparison between group cases.

Abbreviations: FN, false negative; FP, false positive; LB, lower bound; NPV, negative predictive value; PPV, positive predictive value; TN, true negative; TP, true positive; UB, upper bound.

MiR‐1 and miR‐499 were the most sensitive markers (sensitivity 0.917, SCD vs AMI), whereas miR‐208 showed the highest specificity in distinguishing AMI from control cases. In fact, at a cut‐off value of −2.79 (‐ΔCt), 0 false negatives were detected by the test. Overall, miR‐208 and miR‐1 gave the highest values of combined sensitivity + specificity in the comparison of SCD vs AMI cases.

## 4 DISCUSSION

**Figure 5:**
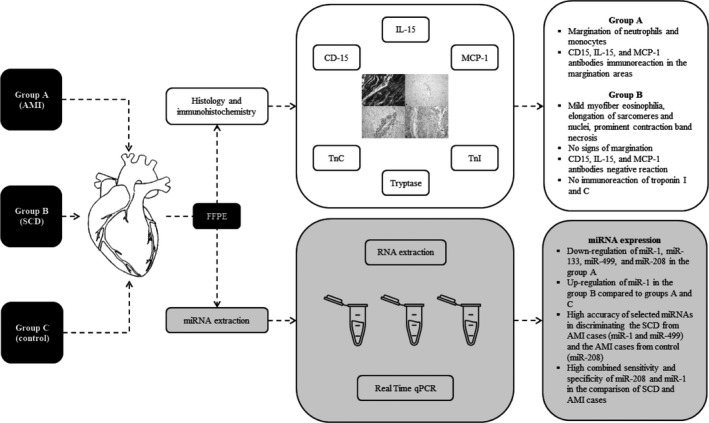
Experimental outline and results

The present study aimed at evaluating the potential diagnostic power of selected miRNAs as tool to differentiate and to quickly orient diagnosis of SCD or AMI. Furthermore, we were interested in proposing miRNAs as candidate markers to improve current diagnosis of SCD or AMI, which is currently performed with the antibody assays present in our work. These antibodies are not the target of selected miRNAs; instead, they represent the current way to discriminate two pathological scenarios. To our knowledge, this study is the first report describing the expression of cardio‐miRNAs comparing AMI to SCD cases (Figure 5). It differs from other investigations that focused on the dysregulation of specific cardio‐miRNAs in AMI.^36^ We investigated the expression of specific cardio‐miRNAs in cardiac tissue samples in subjects deceased for AMI or SCD and whether these different cardiac diseases could be differentiated by the miRNA expression in cardiac tissue samples taken at autopsy. Our results showed that MCP‐1 was strongly expressed in the AMI cases. IL‐15 positivity in AMI cases may be interpreted as an expression of the synergism with neutrophilic granulocytes (CD15), and our study confirms the potential for striking cytokine synergy in promoting the local neutrophil response in damaged tissues.^30^, ^31^, ^32^, ^33^ The demonstrated cardiac IL‐15, MCP‐1 expression in myocardial infarction cases added to confirm a great harvest of data showing that humoural (cytokines and inducible chemokines, complement, and Toll‐like receptors) and cellular (monocytes, macrophages, dendritic cells, T cells, mast cells, platelets, endothelial cells) mediators play a considerable role in the initial healing phases following cardiomyocytes death.^37^ Cx43 immunohistochemically positive reaction demonstrated few brown granules in the AMI group, rarely expressed at the intercellular junctions. Compared with the SCD group, immunolabelled Cx43‐positive granules were small, irregular and scattered. The control group demonstrated Cx43 granules regularly distributed in the intercalated disc and also in the cellular bridges between the myocardial cells as already described.^38^, ^39^

miR‐1 and miR‐499 were the most sensitive markers (sensitivity 0.917, SCD vs AMI), whereas miR‐208 shows the highest specificity in distinguishing AMI from control cases. In fact, at a cut‐off value of −2.79 (‐ΔCt), 0 false negatives were detected by the test. Overall, miR‐208 and miR‐1 gave the highest values of combined sensitivity and specificity in the comparison of SCD vs AMI cases.

miRNAs are only partially complementary to their mRNAs targets.^40^ Consequently, each miRNA can variably control the expression of many genes (up to 200). On the other hand, several miRNAs may cooperate through multiple target sites in one gene. About 30% of all genes are thought to represent miRNA targets,^41^ and it is believed that miRNA genes are 2%‐3% of the total human genes^42^ some of them with a well‐characterized tissue and developmental stage‐specific expression.^43^, ^44^

Given these peculiar miRNAs characteristics, it becomes evident that any dysregulation of the miRNAs may alter the physiological homeostasis thus favouring pathological alterations and manifestations, such as uncontrolled growth or cell proliferation (cardiac hypertrophy or cancer) or electrical dysbalance (SCD).^44^ However, despite the high expression of miRNAs in the heart, the exact understanding of their role is still challenging.

In particular, miR‐1 and miR‐133 are cardiac and skeletal muscle‐specific. miR‐1 is demonstrated to play a key regulatory role in differentiation and proliferation during cardiogenesis and in cardiomyocyte growth in the adult heart while miR‐133, enhancing myoblast proliferation, is a significant factor in cardiomyocyte proliferation. The miR‐208 family, which comprises miR‐208a/b and mi‐499 (exclusively expressed in the heart), are thought to significantly contribute to cardiac hypertrophy and, consequently, arrhythmia. Furthermore, miR‐499 seems to be down‐regulated under pathological conditions such as ischaemia and cardiac remodelling.^45^

Many studies have been performed to characterize those miRNAs involved in AMI.^36^ Yang and colleagues demonstrated that up‐regulation of miR‐1 occurs in structurally diseased human hearts, providing evidence for the role of miR‐1 in electrical remodelling and arrhythmias.^38^ Furthermore, it has been demonstrated that the inhibition of miR‐1 after AMI is able to diminish sudden death in a rat model of surgical myocardial infarction.^46^

The expression of these cardio‐miRNAs has been often evaluated in bodily fluids from subjects who experienced AMI. For example, in a recent study, miRNAs were measured in plasma from subjects who died in the field, died in hospital or survived to discharge. In that study, plasmatic miR‐499‐5p was higher in cases with respect to controls. miR‐499‐5p was lower in both subjects died in hospital and those survived to discharge compared to subjects died in the field. Furthermore, miR‐133, miR‐208 and miR‐499 were lower in subjects survived to discharge compared to those who deceased in the field.^47^, ^48^ In several studies, plasmatic concentrations of miR‐499 were increased in AMI compared to individuals without cardiovascular diseases.^36^ In particular, diagnostic power of miR‐499 in distinguishing AMI reached an AUC of 0.86 [95% CI, 0.81‐0.91], quite lower than troponin [AUC of 0.90 (95% CI, 0.85‐0.95)] but higher than creatine kinase‐MB AUC of 0.82 [95% CI, 0.76‐0.87].^47^ Similarly, serum miR‐1 has been reported to significantly increase in patients with AMI compared to normal controls^48^ and its level was dropped to normal on discharge following medication.^38^ In another study using a rat model, plasmatic miR‐208 increased significantly after isoproterenol‐induced myocardial injury.^49^ Similarly to miR‐499, circulating miR‐1 has also been shown to increase in blood samples from patients experiencing AMI^38^ and a decrease in the infarcted myocardium of a mouse model of AMI was reported. miR‐1 has been proposed as an independent biomarker for the diagnosis of AMI and the associated ischaemic arrhythmias.^38^

In accordance with our results, a recent quantitative analysis assessing microRNA stability in post‐mortem FFPE tissues from subjects dead for AMI compared to healthy controls showed a significant decrease (2.1‐fold) in the expression of miR‐499a.^50^

This fact, together with the increase in miR‐499 in serum from patients experiencing AMI,^51^, ^52^ supports the hypothesis of its release from the cardiac tissue. Furthermore, in vivo models of AMI demonstrated a decrease in miR‐499 levels in the cardiac infarcted zone.^53^ In the same study, a decrease in miR‐1 in AMI tissues compared to controls was found, although not statistically significant.^50^ Contrary to our findings, another study on autoptic infarcted heart tissue from patients with AMI reported an up‐regulation of miR‐208 in these patients compared to healthy adult hearts. However, a down‐regulation of miR‐1 and miR‐133a in AMI patients compared to healthy adult hearts was reported in accordance with our results. In the same study, these expression patterns found in myocardial infarction cases were found to be similar to those from foetal hearts, reinforcing the proposed mechanism of cardiac gene reprogramming in the remodelling of the heart.^54^

Besides their role in cardiac development and in several cardiac pathological processes, such as cardiac hypertrophy, heart failure,^55^, ^56^, ^57^, ^58^ cardiomyopathy^59^ and angiogenesis,^60^ it is believed that several miRNAs are involved in the maintenance of the electrical properties of the heart and that miRNAs imbalance may cause electrical instability and sudden death.^26^, ^54^, ^55^, ^56^, ^57^, ^58^, ^59^, ^60^, ^61^ In fact, many cardiac ion channels are controlled by miRNA at the post‐transcriptional level. The slow‐activating delayed rectifying potassium channel (IKs), the hyperpolarization‐activated (pacemaker) channels (If), the voltage‐gated potassium channel (IKv) and the rapid delayed rectifier potassium current (IKr), the inwardly rectified ion current (IK1), are examples of the cardiac ion channel believed to be under miRNAs control.^44^ miRNA expression is demonstrated to be dynamically regulated and altered miRNA expression can render expression deregulation of ion channel genes leading to channelopathies.^62^, ^63^

Experimental studies showed that muscle‐specific miRNAs as miR‐1 and miR‐133 may influence the spatial patterns of tissue distribution of ion channels.^64^ It has been demonstrated that miR‐133 regulates pacemaker channel HCN2 and HCN4 and contributes to the re‐expression of these channels in hypertrophic heart.^65^, ^66^

In the infarcted area, a significant cytokine up‐regulation is detectable due to different pathways like reactive oxygen species generation, complement activation and NF‐κB (nuclear factor kappa‐light‐chain‐enhancer of activated B cells) activation potently stimulate cytokine mRNA (messenger ribonucleic acid) synthesis in both resident and blood‐derived cells.^67^, ^68^ In infarcted myocardium, an up‐regulation of miR‐1 that down‐regulates the KCNJ2 and GJA1 expressions with the cooperation of miR‐206 is present. The first gene encodes the principle pore‐forming subunit Kir2.1 of the inwardly rectified ion current (IK1), while the second is responsible for connexin 43, a constituent gap junction protein, fundamental for the impulse propagation and electrical synchronization between myocytes.^68^

Our contribution focuses on the diagnostic capacity of miRNAs in discriminating AMI cases from SCD cases, proposing operational difficulties since SCDs are an area that requires more consistent and more robust studies about miRNAs. Actually, the expression of specific cardio‐miRNAs in cardiac tissue samples in subjects deceased for AMI or SCD could be differentiated by the miRNA expression in cardiac tissue samples taken at autopsy.^69^ The study of cardio‐miRNAs expression may improve clinicians and pathologists’ understanding of the mechanisms underlying to cardiac insults and provide targets for future therapies. As has been pointed out, “Those who are working in this field would be well advised to… focus on opportunities where there is a lower barrier of entry for miRNA‐based diagnostics, and then perform robust and reproducible studies using biologically applicable samples. Only then will miRNAs succeed as a class of biomarkers for cardiovascular disease”.^50^

Further studies should be carried out with this specific research purpose.

## CONFLICT OF INTEREST

The authors confirm that there are no conflicts of interest.

## AUTHORS CONTRIBUTION

All the authors listed contributed to the research study design and performed data collection and analysis and interpretation of data. All authors drafted the paper or revised it critically, and they approved the submitted and final versions.

## Data Availability

The data that support the findings of this study are available on request from the corresponding author. The data are not publicly available due to privacy or ethical restrictions.
